# Ultrasonography Evaluation of Umbilical Structures in Clinically Healthy Donkey Foals during the First Week of Life

**DOI:** 10.3390/ani11061650

**Published:** 2021-06-02

**Authors:** Valentina Vitale, Irene Nocera, Micaela Sgorbini, Benedetta Aliboni, Fulvio Laus, Aurora Mannini, Marilena Bazzano

**Affiliations:** 1 Department of Veterinary Sciences, Viale Delle Piagge 2, 56124 Pisa, Italy; v.vitale_vet@yahoo.es (V.V.); micaela.sgorbini@unipi.it (M.S.); benedetta.aliboni@libero.it (B.A.); aurora.mannini@gmail.com (A.M.); 2 Veterinary Teaching Hospital, Via Livornese Snc, San Piero a Grado, 56122 Pisa, Italy; 3 School of Biosciences and Veterinary Medicine, University of Camerino, 62032 Camerino, Italy; fulvio.laus@unicam.it (F.L.); marilena.bazzano@unicam.it (M.B.)

**Keywords:** donkey, foal, umbilicus, ultrasound

## Abstract

**Simple Summary:**

Umbilicus assessment is an essential part of the clinical examination in neonates because the umbilicus could be a potential access-point for pathogens. Thus, an early and accurate diagnosis is pivotal in order to check for any evidence of umbilical infections and potentially life-threating sepsis. Ultrasound scanning has been shown to be a valuable tool, both in equine foals and calves, in order to assess the diameter and appearance of external and intra-abdominal umbilicus remnant. To the best of our knowledge, no reports are available for donkey foals; thus, the present study evaluates ultrasonography and measures the umbilical remnants in donkey foals in the first week of life. No statistical differences in the size and appearance of the umbilical remnants were observed during the first week of life in the donkey foals, and the measurement values reported in this study are comparable with the ones obtained in equine foals and calves.

**Abstract:**

The umbilicus is a potential access-point for pathogens in equine foal, causing umbilical infections and potentially life-threating illness. Early diagnosis based on ultrasonographic appearance and measurement is crucial to avoid severe complications and promptly implement appropriate therapy. This study ultrasonographically evaluates the umbilical remnants of donkey foals, in the first week of life. Fifteen healthy donkey foals were included in the study. The umbilical vein, arteries and urachus ultrasounds were performed at 24 h, 3 and 7 days of life, using a portable ultrasound machine and a 5–7.5 MHz multifrequency linear probe. The Kruskal–Wallis test and Dunn’s multiple comparisons test were applied to verify differences in relation to time for all the umbilical remnants measured. Statistical significance was set at *p* < 0.05. No statistical differences were observed in relation to time regarding umbilical remnant measurements. A correlation was found between body weight and the left artery at T0. The regression of the umbilical remnant during the first week of life was slower compared with what was reported in equine foals but was comparable with the results on calves. Thus, the different regression timing needs be considered when evaluating donkey foals with umbilical remnant diseases within the first week of life.

## 1. Introduction

Donkeys (*Equus asinus*) represent an important percentage of the world equine population [1]. They have been close companions of humans for millennia and are used as working animals. Nowadays, donkeys are also used for milk production and animal-assisted therapy [1] and are considered pets [2]. For these reasons, the importance of knowing the most common diseases of this species has increased. The scientific literature has shown a renewed interest in these animals regarding their welfare [3], infectious diseases [4,5,6,7], the need for specific diagnostic criteria and reference values in adult donkeys [6,7,8,9,10,11,12,13,14], pregnant and lactating jennies [13,14,15,16] and donkey foals [17,18,19].

In foals, the umbilical remnants diseases (i.e., infection or patent urachus) are considered a relevant problem for the neonate [20,21,22,23,24], and an early diagnosis is of great importance [21,23,24,25,26]. Moreover, umbilical remnants could be a potential access-point for pathogens, causing infectious diseases, sepsis and neonatal mortality [27,28]. Ultrasonography represents a useful and valuable diagnostic tool in equine practice and is in common use by veterinarian practitioners, both in field and hospital settings. The literature on the umbilical cord ultrasonography appearance of equine foals is abundant [29,30]. The use of high-frequency linear (10–12 MHz) or transrectal (5–7.5 MHz) probes has been suggested, and the hair should be clipped, whenever possible, from the xiphoid caudal to the inguinal region, wetting with alcohol and coupling gel [23]. Horse foals tolerate the ultrasound procedure well without the need for sedation; moreover, this procedure can be performed while the foal is either standing beside the mare or in lateral recumbency, properly restrained [23,26]. The following structures are usually scanned during the procedure: the external umbilical remnant, umbilical vein, urachus and umbilical arteries [23]. The most common pathologies that can be detected with the use of ultrasound are infection of the external umbilical remnant, vein, arteries or urachus; umbilical hernia; and patent urachus [23].

The umbilical remnants in calves used to be checked by clinical examination of the umbilicus area, but an ultrasonographic technique has also been described. The ultrasound scan has been described as an accurate imaging technique for external and intra-abdominal umbilical remnant size and appearance [31,32]. This procedure has been described as being performed while the calf is standing in a similar way as in the equine foal, using a 5 MHz linear probe [31,32].

To the authors’ knowledge, no ultrasound appearance description, size reference values and physiological atrophy time can be found on umbilicus remnants in donkey foals. Thus, the aims of this study were (1) to describe the umbilical remnant ultrasonographic examination in healthy donkey foals in the first week of life and (2) to report the measurements of the umbilical structures in these foals.

## 2. Materials and Methods

### 2.1. Animals

A total of fifteen healthy donkey foals were included in this research. Nine out of 15 were Amiata donkey foals (6 fillies and 3 colts) born at the Department of Veterinary Sciences, University of Pisa during the 2012 and 2019 foaling seasons, and 6/15 (3 fillies and 3 colts) were mixed-breed donkey foals born at the stud farm, Azienda Agricola “Cambiotti”, in Perugia Province, during the 2020 foaling season. Foals were considered healthy on the basis of their APGAR score and a clinical examination performed at birth, and then each day until 7 days of age. The external umbilical stump was also visually assessed. Foals were weighed immediately after standing, using a dedicated scale, and were housed in a box with the mare for the entire study-period.

Approval to conduct this study was obtained from the Ethics Committee on Animal Experimentation of the University of Pisa and transmitted to the Italian Ministry of Health for the 2012 foaling season, in line with the D.Lgs 116/92. The 2019 and 2020 foaling seasons were approved by the “Organismo Preposto al Benessere Animale” (OPBA), University of Pisa, according to the D.Lgs. 26/14 (Prot. N. 33476/16).

### 2.2. Ultrasonography Technique

**Figure 1:**
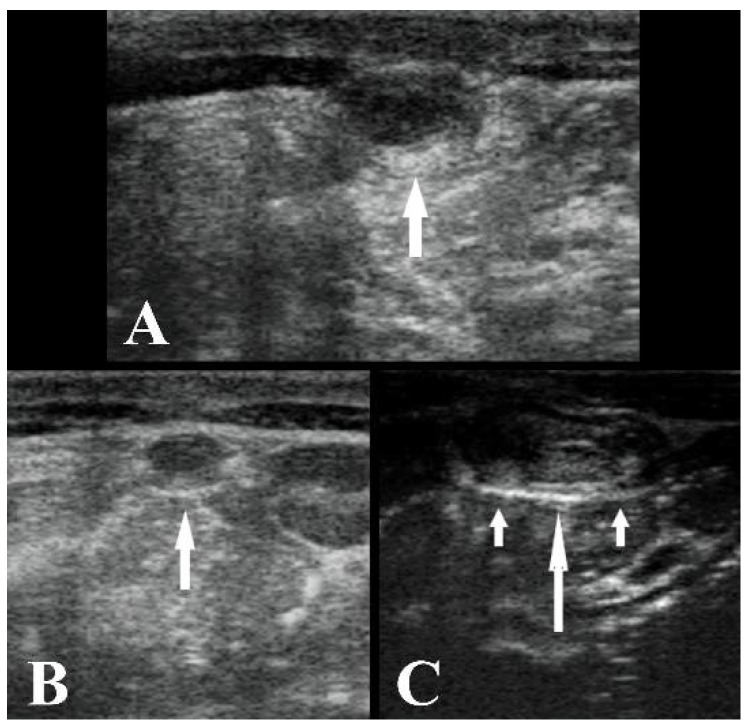
(**A**) Ultrasound image of the umbilical vein cranial to the umbilical stump at T0 (white arrow), transverse section. (**B**) Ultrasound image of the umbilical vein at the xiphoid cartilage at T0 (white arrow), transverse section. (**C**) Ultrasound image of the urachus (big white arrow) and left and right arteries (small white arrows) at T0, transverse section. The left side of the images is left, and the right side is right. B-mode, linear probe 7.5 MHz.

**Figure 2:**
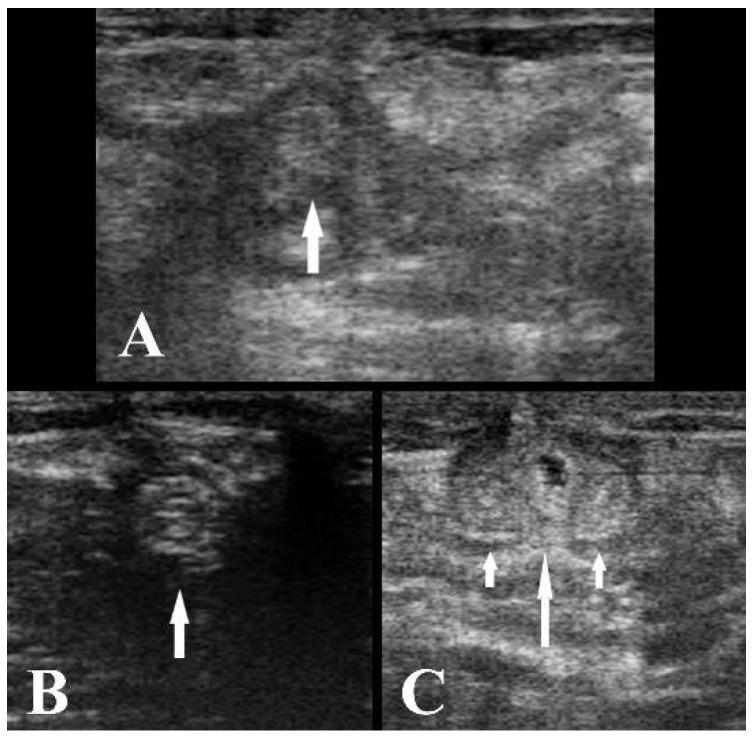
(**A**) Ultrasound image of the umbilical vein cranial to the umbilical stump at T1 (white arrow), transverse section. (**B**) Ultrasound image of the umbilical vein at the xiphoid cartilage at T1 (white arrow), transverse section. (**C**) Ultrasound image of the urachus (big white arrow) and left and right arteries (small white arrows) at T1, transverse section. The left side of the images is left, and the right side is right. B-mode, linear probe 7.5 MHz.

**Figure 3:**
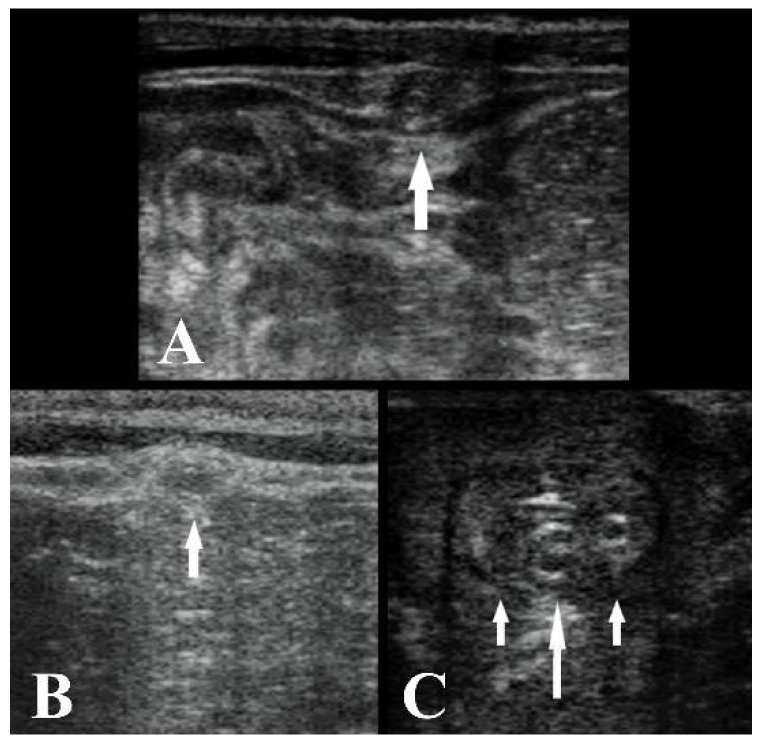
(**A**) Ultrasound image of the umbilical vein cranial to the umbilical stump at T2 (white arrow), transverse section. (**B**) Ultrasound image of the umbilical vein at the xiphoid cartilage at T2 (white arrow), transverse section. (**C**) Ultrasound image of the urachus (big white arrow) and left and right arteries (small white arrows) at T2, transverse section. The left side of the images is left, and the right side is right. B-mode, linear probe 7.5 MHz.

The ultrasound evaluation of the umbilical remnant was performed at 24 h (T0) (Figure 1) and then again at 3 (T1) (Figure 2) and 7 (T2) (Figure 3) days of life. The ultrasound examination was performed in lateral recumbency (right or left) in the presence of the dam by two experienced operators (MS and FL). None of the foals were sedated, but they were manually restricted. No hair clipping was performed, and only alcohol coupled with ultrasound gel was applied to provide appropriate contact [23,26].

Ultrasound was performed with a real-time B mode scan using a portable ultrasound machine (MyLab30Gold, Esaote, Italy) and a 5–7.5 MHz multifrequency linear probe transducer [21,23,24,25,26,33,34]. The following anatomic structures were examined: umbilical arteries caudal to the external stump, the urachus and the urachus–arteries complex at the apex of the bladder, and the umbilical vein, which was scanned from just cranial to the external stump to caudal to the xiphoid cartilage [23,25,26,34].

Both short (transverse) and long-axis (sagittal) views were obtained for all the structures in order to carried out a qualitative evaluation, while the measurements were evaluated on the transverse view. The transverse view was obtained by placing the probe with an orientation perpendicular to the lumbar spine, while the sagittal view was obtained by placing the probe parallel to the lumbar spine [26,34]. The ultrasound exam of the vein was performed in a caudo-cranial direction, from the umbilical stump to the liver [23,25,34], while the other structures were examined in a cranio-caudal direction from the umbilical stump to the bladder [26].

The ultrasound video and images were recorded, and the qualitative evaluation and measurements of each umbilical structure were performed offline using dedicated software (MyLab Desk, Esaote, Italy). The measurements were performed by 3 experienced operators (VV in 2012; IN in 2019; MB in 2020). The measurements of greatest cross-sectional diameter were performed on the transverse view, and they were evaluated on the following areas: for the umbilical vein just cranial to the external umbilicus (“cranial umbilicus”) and at the level of the xiphoid cartilage (“xiphoid cartilage”), and for urachus and arteries, just caudal to the external umbilicus.

### 2.3. Statistical Analysis

Data were analyzed for distribution using the Shapiro–Wilk test. Since data did not show a Gaussian distribution, results were expressed as median, minimum and maximum values. The Mann–Whitney test was applied to verify differences in body weight (BW) between the two groups (Amiata versus mixed breed) and between sexes. The Kruskal–Wallis test and Dunn’s multiple comparisons test were applied to verify differences in relation to time for all the umbilical remnant structures measured. The Spearman test was performed to verify the correlation between BW and measures recorded at all times. Statistical significance was set at *p* < 0.05.

## 3. Results

The umbilical stump was visually normal at birth in all the included donkey foals.

The median BW was 26.75 (22.5–34.5 kg) and 25 kg (25–35 kg) in Amiata donkey foals and in mixed-breed foals, respectively. In fillies, the median BW was 25 kg (22.5–34.5 kg), while in colts, it was 29 kg (25–35 kg). No differences in BW were found between the two groups (Amiata versus mixed breed) (*p* = 0.5515) or between fillies and colts (*p* = 0.2044).

The measurements of the umbilical structures recorded in donkey foals were reported in Table 1. No statistical differences were observed in relation to time for the right (*p* = 0.8223) or left (*p* = 0.6011) arteries, the umbilical vein (*p* = 0.8758), the urachus (*p* = 0.2757) or the urachus–arteries complex (*p* = 0.1683).

A correlation was only found between BW and left artery measurement at T0 (r = 0.5453; *p* = 0.0461).

## 4. Discussion

This study describes, for the first time, the ultrasonography of umbilical structures in donkey foals. The umbilical cord is composed, in both equine and donkey foals, of the amniotic sheath, two arteries, the urachus and the umbilical vein [1,20,23,24,27,34]. The vein courses along the midline, cranially to the liver, very close to the skin surface, until the xiphoid process, and then it bends dorsally and enters the liver. After parturition, it atrophies and becomes the round ligament of the liver, part of the falciform ligament [1,23,26]. The umbilical arteries branch from the internal pudendal artery and course along the sides of the bladder before entering the umbilical stump. The arteries also atrophy postpartum and become the round ligaments of the bladder [1,23,26]. Fetal urine is excreted through the urachus, which connects the apex of the bladder to the allantoid cavity. The urachus courses between the umbilical arteries that close and atrophy along their entire length after birth [1,23,26].

In this study, the measurements of arteries, vein and urachus did not differ from those reported for equine foals [21,30] and also for Holstein calves, both at 24 h and 7 days [31,32,35]. This is surprising because the size of anatomical structures is usually related to body size, as has been shown for the front digit structures, comparing horses and donkeys [10]. A neonatal Amiata donkey foal weighs approximately 27.83 ± 5.40 kg, which is 2/3 the weight of a typical standardbred foal at birth [12], and a similar weight has also been found in the mixed-breed group; moreover, this value is lower than the mean body-weight value reported in Holstein newborns, which is 34 kg [35]. Nevertheless, the mean length of the umbilical cord recorded in donkeys was similar to that described in horses [1]; thus, it is possible that the proportions for these structures do not have similar correlation with BW.

We did not find significative differences in the measures of the umbilical cord structures between fillies and colts, in line with what has been shown both in foals [30] and calves [32]. Moreover, this result might be correlated and supported by the absence of differences even for BW, as reported previously [14].

Another interesting finding is that the percentage of reduction in size of the umbilical remnants described in standardbred foals is approximately 20% at one week of age [30]. We did not observe any change in size of the structures measured over the seven days of observation; thus, no regression of the umbilicus was found during the first week of age in donkey foals. In healthy calves, it has been shown that there is no significative change within one week of life in the size of the umbilical vein and arteries; however, they substantially decreased from day 1 to four weeks of age [31,32,35]. It is possible that the atrophy of the umbilical arteries and vein is slower in donkeys compared to horses, and it might be significative after a prolonged observation period, as shown in calves. Thus, more studies are warranted in order to assess the onset, rate and duration of regression.

Finally, the only correlation found between BW and the umbilical structures at T1 was with the left umbilical artery. This result could be related to the small size of the population enrolled, or it could be a type 1 statistical error; thus, further studies including a large number of donkey foals are needed to evaluate the relation between BW and the measures of the umbilical structures.

This study has some limitations, such as a low number of animals included and that two different donkey breeds were enrolled. Several breeds of donkey exist around the globe with different body-size; thus, it could possible that the values obtained in this paper may not apply to other breeds. Moreover, the foals were judged to be healthy based only on a physical exam. As they did not develop any disease even after the study was performed, it is unlikely that they were affected by any umbilical abnormality, but this cannot be completely excluded. Finally, as already mentioned, the ultrasonography was performed from birth until one week of age; thus, we cannot describe the timing of regression of umbilical remnants in donkeys.

## 5. Conclusions

Contrary to what has been found for hematological and biochemical parameters [8], the ranges of umbilical measurements, including the urachus, vein and arteries, reported for horses were comparable with what has been found in donkeys for the first week of age. The regression of the structures is probably slower compared with equine foals, and this should be taken into account when evaluating older foals with possible umbilical disease. Further studies are needed in order to establish the onset and rate of involution during the first months of life in donkey foals.

## Figures and Tables

**Table 1:** Measurements obtained for the umbilical structures in donkey foals at birth (T0), three days (T1) and one week (T2). Values are reported in cm and expressed as median (minimum–maximum value). The measurements are reported for the umbilical vein (UV) just cranial to the external umbilicus (“cranial umbilicus”) and at the level of the xiphoid cartilage (“xiphoid cartilage”).

Parameters	T0 cm	T1 cm	T2 cm
Right Artery	0.63 (0.44–1.58)	0.68 (0.44–1.77)	0.73 (0.34–1.72)
Left Artery	0.71 (0.43–1.96)	0.72 (0.53–1.70)	0.73 (0.46–1.90)
Urachus	0.61 (0.46–1.12)	0.87 (0.58–2.46)	0.78 (0.55–3.30)
Urachus–Arteries Complex	2.21 (1.86–3.46)	2.16 (0.90–3.01)	2.24 (0.54–3.01)
UV cranial umbilicus	0.89 (0.47–1.83)	0.73 (0.30–2.01)	0.79 (0.34–1.87)
UV xiphoid cartilage	0.88 (0.52–1.72)	0.67 (0.42–2.11)	0.62 (0.31–1.87)

## Data Availability

The data presented in this study are available on request from the corresponding author.

## References

[1] Carluccio A., Panzani S., Tosi U., Riccaboni P., Contri A., Veronesi M.C. (2008). Morphological features of the placenta at term in the Martina Franca donkey. Theriogenology.

[2] El-Shafaey E.A., Salem M.G., Mosbah E., Zaghloul A.E. (2017). Morphometric evaluation of relevant radiographic parameters of the forefeet of clinically normal donkeys (Equus asinus). J. Hell. Vet. Med. Soc..

[3] Rota A., Sgorbini M., Panzani D., Bonelli F., Baragli P., Ille N., Gatta D., Sighieri C., Casini L., Maggiorelli M.M. (2018). Effect of housing system on reproductive behaviour and on some endocrinological and seminal parameters of donkey stallion. Reprod. Dom. Anim..

[4] Barrandeguy M.E., Carossino M. (2018). Infectious diseases in donkeys and mules: An overview and update. J. Equine Vet. Sci..

[5] Sgorbini M., Veronesi F., Fratini M., Laus F. (2018). Tick-borne diseases and gastric ulcer in the donkey. J. Equine Vet. Sci..

[6] Papini R.A., Orsetti C., Sgorbini M. (2020). A controlled study on efficacy and egg reappearance period of Ivermectin in donkeys naturally infected with small strongyles. Helminthologia.

[7] Papini R.A., Orsetti C., Sgorbini M. (2020). Evaluation of a marked polyherbal dewormer against intestinal strongyles in naturally infected donkeys. Helminthologia.

[8] Bonelli F., Rota A., Aurich C., Ille N., Camillo F., Panzani D., Sgorbini M. (2019). Determination of salivary cortisol in donkey stallions. J. Equine Vet. Sci..

[9] Bonelli F., Nocera I., Conte G., Panzani D., Sgorbini M. (2019). Relation between APGAR scoring and physical parameters in 44 newborn Amiata foals at birth. Theriogenology.

[10] Bazzano M., McLean A., Tesei B., Gallina E., Laus F. (2019). Selenium and Vitamin E Concentrations in a Healthy Donkey Population in Central Italy. J. Equine Vet. Sci..

[11] Nocera I., Aliboni B., Puccinelli C., Pietrini G., Sgorbini M., Citi S., Ricardi G. (2020). Radiographic parameters of the digit in a cohort population of Amiata donkeys. Open Vet. J..

[12] Nocera I., Aliboni B., Sgorbini M., Gracia-Calvo L.A., Conte G., Ben David L., Citi S. (2020). Ultrasonographic appearance of elbow joints in a population of Amiata donkeys. J. Equine Vet. Sci..

[13] Turini L., Bonelli F., Nocera I., Meucci V., Conte G., Sgorbini M. (2021). Evaluation of different methods to estimate the transfer of immunity in donkey foals fed with colostrum of good IgG quality: A preliminary study. Animals.

[14] Turini L., Nocera I., Bonelli F., Mele M., Sgorbini M. (2020). Evaluation of Brix refractometry for the estimation of colostrum quality in Jennies. J. Equine Vet. Sci..

[15] Crisci A., Rota A., Panzani D., Sgorbini M., Ousey J.C., Camillo F. (2014). Clinical, ultrasonographic, and endocrinological studies on donkey pregnancy. Theriogenology.

[16] Bonelli F., Busechian S., Meucci V., Caporrino G., Briganti A., Rueca F., Zappulla F., Ferini E., Ghiandai L., Sgorbini M. (2016). pHyloGASTRO in the treatment of Equine Gastric Ulcer lesions. J. Equine Vet. Sci..

[17] Carluccio A., Noto F., Parrillo S., Contri A., De Amicis I., Gloria A., Robbea D., Veronesi M.C. (2016). Transrectal ultrasonographic evaluation of combined utero-placental thickness during the last half of pregnancy in Martina Franca donkeys. Theriogenology.

[18] Sgorbini M., Bonelli F., Rota A., Baragli P., Marchetti V., Corazza M. (2013). Hematology and clinical chemistry in Amiata donkey foals from birth to 2 months of age. J. Equine Vet. Sci..

[19] Veronesi M.C., Gloria A., Panzani S., Sfirro M.P., Carluccio A., Contri A. (2014). Blood analysis in newborn donkeys: Hematology, biochemistry, and blood gases analysis. Theriogenology.

[20] Carluccio A., Contri A., Gloria A., Veronesi M.C., Sfirro M.P., Parrillo S., Robbe D. (2017). Correlation between some arterial and venous blood gas parameters in healthy newborn Martina Franca donkey foals from birth to 96 hours of age. Theriogenology.

[21] Lavan R.P., Craychee T., Madigan J.E. (1997). Pratical method of umbilical ultrasonographic examination of one-week old foals: The procedure and the interpretation of age-correlated size range of umbilical structures. J. Equine Vet. Sci..

[22] Magata F., Ishii M., Oikawa E., Furuoka H., Yamada K., Sasaki N., Shimizu S., Inokuma H. (2010). Purulent necrotic dislocation of the hip joint associated with umbilical infection in a foal. J. Equine Sci..

[23] Magri M., Kidd J.A., Lu K.G., Frazer M.L. (2014). Ultrasonography of umbilical structures. Atlas of Equine Ultrasonography.

[24] Magri M. (2018). Ultrasonography of the umbilical remnant in foals. Practice.

[25] Reef V.B., Collatos C. (1998). Ultrasonography of umbilical structures in clinically normal foals. Am. J. Vet. Res..

[26] Franklin R.P., Ferrell E.A. How to perform umbilical sonograms in the neonate. Proceedings of the Annual Convention of the AAEP.

[27] Elce Y.A. (2006). Infections in the Equine abdomen and pelvis: Perirectal abscesses, umbilical infections and peritonitis. Vet. Clin. Equine.

[28] Ortega J., Daft B., Assis R.A., Kinde H., Anthenill L., Odani J., Uzal F.A. (2007). Infection of internal umbilical remnant in foals by Clostridium sordelli. Vet. Pathol..

[29] Corley K., Corley K., Stephen J. (2008). Procedures in the neonatal foal. The Equine Hospital Manual.

[30] McCoy A.M., Lopp C.T., Kooy S., Migliorisi A.C., Austin S.M., Wilkins P.A. (2020). Normal regression of the internal umbilical remnant structures in Standardbred foals. Equine Vet. J..

[31] Watson E., Mahaffey M.B., Crowell W., Selcer B.A., Morris D.D., Seginak L. (1994). Ultrasonography of the umbilical structures in clinically normal calves. Am. J. Vet. Res..

[32] Guerri G., Vignoli M., Palombi C., Monaci M., Petrizzi L. (2020). Ultrasonographic evaluation of umbilical structures in Holstein calves: A comparison between healthy calves and calves affected by umbilical disorders. J. Dairy Sci..

[33] Wilkins P.A., Reed S.M., Bayly W.M., Sellon D.C. (2017). Disorders of foals. Equine Internal Medicine.

[34] Sprayberry K.A. (2015). Ultrasonographic examination of the equine neonate: Thorax and abdomen. Vet. Clin. Equine.

[35] Lischer C.J., Steiner A. (1993). Ultrasonography of the umbilicus in calves. Part 1: Ultrasonographic description of umbilical involution in clinically healthy calves. Schweiz Arch. Tierheilkd..

